# Temporary pacemaker insertion for severe bradycardia following pneumoperitoneum during robot-assisted radical prostatectomy: a case report

**DOI:** 10.1186/s12893-020-00902-9

**Published:** 2020-10-14

**Authors:** Fumito Yamabe, Yozo Mitsui, Orie Hoshino, Tomo Shimizu, Mizuki Kasahara, Hideyuki Kobayashi, Koichi Nakajima

**Affiliations:** Department of Urology, Faculty of Medicine, Toho University, Tokyo, 143-8540 Japan

**Keywords:** Robot-assisted radical prostatectomy (RARP), Bradycardia, Pneumoperitoneum, Temporary pacemaker

## Abstract

**Background:**

Pneumoperitoneum to maintain a constant gas flow to assist various surgeries is known to cause severe bradycardia and has been linked to heart failure;; however, a recent study demonstrated that it is not linked to poorer surgical outcomes; accordingly, it does not require routine preventive measures. Thus, whether there is a link between sudden bradycardia development and surgical procedures is controversial. We report the case of severe bradycardia that occurred along with a complete atrioventricular block (CAVB) during peritoneum creation in robot-assisted radical prostatectomy (RARP).

**Case presentation:**

A 72-year-old man presented at our hospital with prostate cancer and underwent RARP. After pneumoperitoneum, severe bradycardia and CAVB were observed; thus, the surgery was extended by inserting a temporary pacemaker (TPM).

**Conclusion:**

Because of the difficulty in performing emergency procedures in robot-assisted surgeries, the current case is reported to provide an awareness that surgeons should be cautious of the possible complication of bradycardia and CAVB during such operations, and thus should take steps necessary for managing induction of such conditions.

## Background

A report has indicated the existence of bradycardia during operation in various surgical fields because of vagal reflex caused by surgical stimulation or drugs [1]. Bradycardia during pneumoperitoneum manipulations is considered a sign for predicting unexpected cardiac arrest [2], and thus requires urgent measures and treatment. In fact, the incidence of cardiac arrest during laparoscopic surgery ranges from 0.002 to 0.02% [3]. However, a recent report indicated no possibility of bradycardia and subsequent risk of cardiac arrest associated with poorer surgical outcomes, and hence ruling out the requirement for routine prevention [4]. In this study, we treated a patient who exhibited severe bradycardia with a complete atrioventricular block (CAVB) during robot-assisted radical prostatectomy (RARP). The surgery was extended by insertion of a temporary pacemaker (TPM), which we report along with a review of related cases in the literature.

## Case presentation

**Fig. 1:**
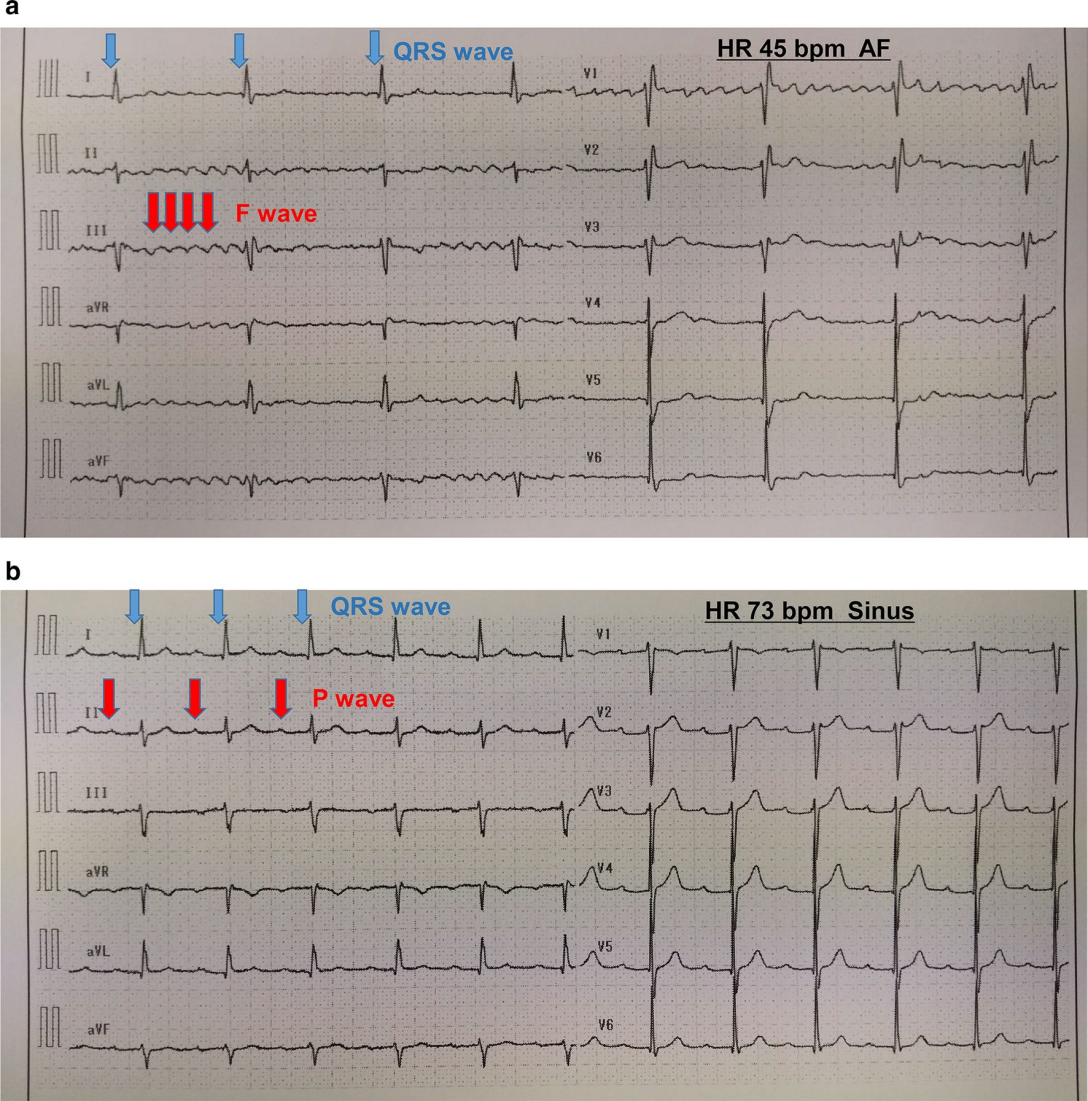
Preoperative 12-lead electrocardiography. **a** Paroxysmal atrial flutter (P-AFL) is present, **b** first-degree atrioventricular block is present

**Fig. 2:**
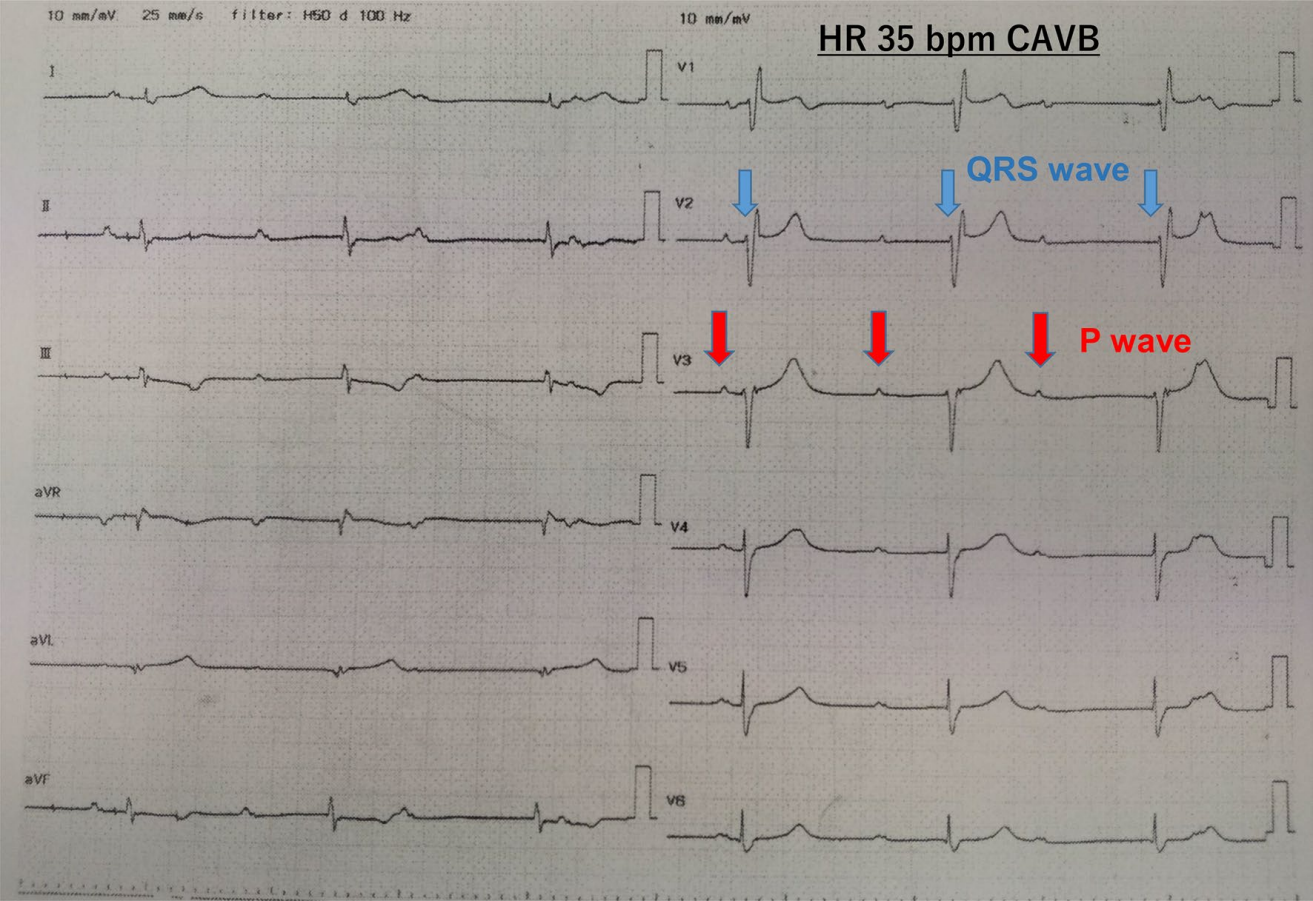
Intraoperative 12-lead electrocardiography HR 35 bpm. A complete atrioventricular block (CAVB) is present

**Fig. 3:**
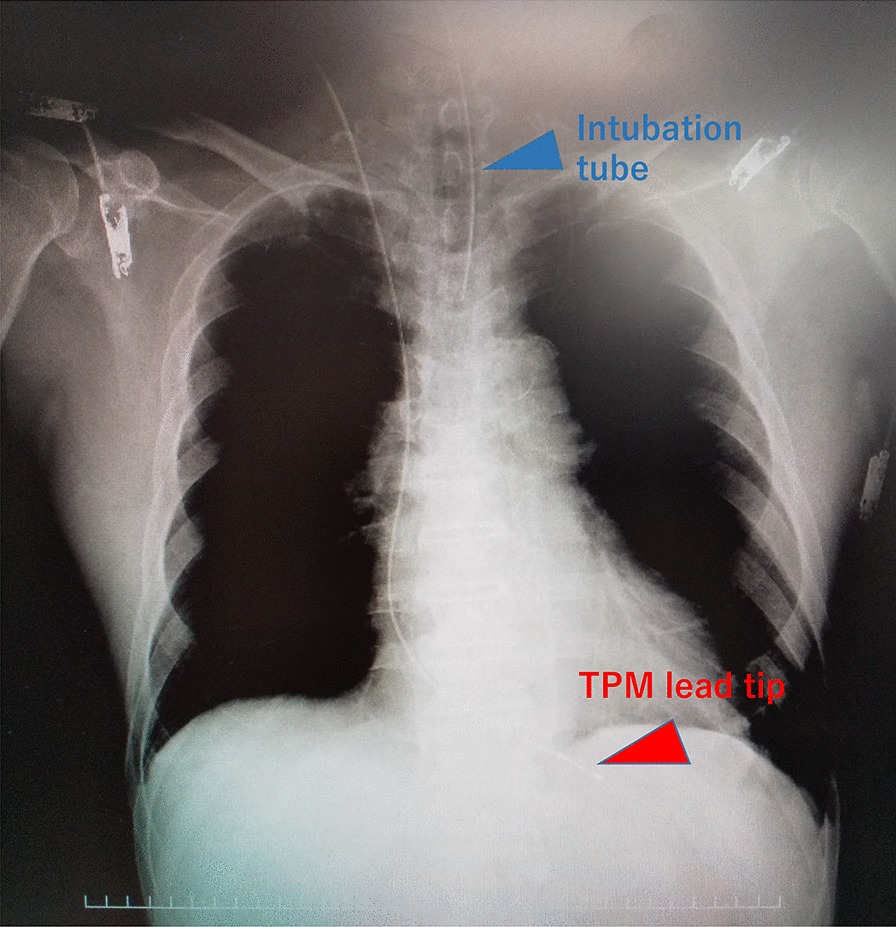
Intraoperative chest X-ray image after temporary pacemaker (TPM) insertion. There is an inserted pacemaker lead

The patient was a 72-year-old man (height: 163 cm, weight: 69 kg) who presented at our facility with a diagnosis of cT2N0M0 prostate cancer. He was being treated at the department of cardiology for hypertension on an outpatient basis with paroxysmal atrial flutter (P-AFL) (Fig. 1a), for which he was taking oral calcium antagonists and direct oral anticoagulants. A first-degree atrioventricular block had been previously identified (Fig. 1b) with no subjective symptoms. Preoperative echocardiography revealed good cardiac function with no valvular disease. Under general anesthesia, RARP was performed using the da Vinci X® Surgical System (Intuitive Surgical, Sunnyvale, CA, USA). Pneumoperitoneum was commenced at a CO_2_ insufflation rate of 1L/min, which was changed to ~ 40 L/min ~ 1 min later. Intra-abdominal pressure was set to 10 mmHg. Then, systolic blood pressure was maintained at ≥ 100 mmHg. However, the patient's heart rate dropped to 30 bpm, indicating bradycardia, and on the electrocardiography monitor, CAVB was observed (Fig. 2). After lacing the ports for the robot-assisted surgery and adopting the Trendelenburg position of 22°, the patient developed bradycardia. Therefore, pneumoperitoneum was stopped and the patient was returned to a horizontal position. First, transcutaneous pacing was performed to temporarily enhance the heart rate because of the observed medical emergency. When the pacing was switched off, spontaneous circulation disappeared (for ≥ 3 s), which compelled us to recommence the pacing immediately and to perform pacing more reliably by inserting a TPM via the right internal jugular vein (Fig. 3), thus conducting the pacing at VVI set rate of 60 bpm. The patient's sustained stable hemodynamics with the TPM. Upon obtaining informed consent from the subject's family, surgery was performed as planned (operation duration: 313 min, duration of pneumoperitoneum: a total of 175 min, blood loss: 50 ml, and resected specimen: 100 g). After the surgery, while continuing to monitor our patient by electrocardiography, the pacing was gradually withdrawn. Electrocardiography performed on postoperative day 2 revealed no CAVB. Then, the patient's heart rate did not go below 40 bpm, and coronary angiography (CAG) performed on postoperative day 6 showed no predominant lesion. Accordingly, the Department of Cardiology deemed the insertion of the permanent pacemaker to be unnecessary; thus, the TPM was removed, and on postoperative day 9, the subject was discharged, as planned. The radical prostatectomy specimen was then diagnosed as adenocarcinoma with Gleason Score 3 + 4 and pT2c EPE0 RM0. At present, one year after surgery, there has been no recurrence of prostate cancer, and the subject is progressing without any arrhythmia-related symptoms.

## Discussion and conclusions

Pneumoperitoneum affects hemodynamics and generally increases the mean arterial pressure, systemic vascular resistance, pulmonary vascular resistance, heart rate, and central venous pressure, but reduces venous return and cardiac output [5]. The incidence of arrhythmia because of pneumoperitoneum is said to range from 14 to 27% [3], the majority of which is sinus tachycardia and extrasystole. However, on rare occasions, severe bradycardia (sinus bradycardia and atrioventricular conduction disturbance) is presented, which is considered to be attributed to a vagal reflex. The reported causes underlying the onset of an excessive vagal nerve response include peritoneal stretching, attributable to pneumoperitoneum, and intraoperative manipulations involving organs within the abdominal cavity [5, 6]. In laparoscopic radical prostatectomy, Trendelenburg positioning under general anesthesia might cause severe bradycardia [7]. In our present case, immediately after the start of pneumoperitoneum, bradycardia and CAVB were observed, the cause of which was considered to be a vagus nerve response to peritoneal stretching. Note that highly frequent causes of CAVB are said to include ischemic heart disease and cardiomyopathy. However, the CAG performed after the surgery revealed no explicit results. In terms of peritoneal stretching because of pneumoperitoneum, when the rate of CO_2_ insufflation starts at a slow rate, it is unlikely that a vagus reflex would occur [8]. Moreover, in the present case, it is possible that if the CO_2_ insufflation rate was increased over a longer period, then the excessive vagus reflex could have been avoided. Typically, in the event of sinus bradycardia, atropine administration is considered. Nevertheless, in our present case, CAVB was observed; hence, we decided to treat the patient by pacing. In RARP, the patient is docked to a patient cart during surgery. When surgery is interrupted to administer respiratory or circulatory emergency procedures, the cart must be detached from the patient, which makes an immediate response more difficult, compared with regular laparoscopic surgery routine. Therefore, we completed the surgery after inserting a TPM, which enabled more reliable pacing than transcutaneous pacing and a favorable outcome.

To the extent of our literature search, this is the first report of surgery performed after inserting a TPM for bradycardia development during RARP. In surgery using pneumoperitoneum, severe bradycardia can, on rare occasions, cause cardiac arrest and therefore requires proper treatment. In robot-assisted surgery, it is important to have countermeasures for complications because it is difficult to interrupt surgery and immediately perform emergency procedures.

## Data Availability

The datasets used during this study available from the corresponding author on reasonable request.

## Abbreviations

CAG: Coronary angiography
CAVB: Complete atrioventricular block
P-AFL: Paroxysmal atrial flutter
RARP: Robot-assisted radical prostatectomy
TPM: Temporary pacemaker
